# When Is “Early” in Operating for Appendicitis?

**Published:** 1907-01

**Authors:** 


### WHEN IS "EARLY" IN OPERATING FOR APPENDICITIS?

A. M. Pond, in the *Medical Record*, argues that the sequence and relation of the symptoms, and not time, should be tbe determining factor in settling this much-asked question. He says that the first symptom to appear, and the first symptom of importance, is the pain, the character and the location of which may well be studied, because it has to do directly with the pathology of the part; it owes its very presence to a diseased condition of the organ, and frequently there is a very close relation existing between the pain and the degree of disturbance; the more severe the pain the more grave the pathology.

This is only true, however, of the appendix that remains intact, for as soon as a perforation occurs, the distended appendix is relieved and the pain ceases; the absence of pain, then, is certainly not a good omen.

The character of this important symptom is at first colicky, and as a rule recurs periodically; the location is at first at or near the umbilicus, and after a few spasms of pain nausea or the second cardinal symptom becomes conspicuous. The pain gradually shifts into the lower portion of the abdomen, usually into the right fossa, but sometimes into the left, and rarely to a point above the bladder. As the pain becomes more constant it usually becomes more intense, and as its intensity increases the nausea is more frequent and more severe; with the increasing pain and nausea there is usually a local peritonitis that can first be detected by percussion of the right fossa; a distinct spasm of the right rectus can be felt before any marked rigidity can be detected by gradual pressure. The symptoms, then, can be classified according to their occurrence, and they occur in exact relation with their importance. Pain, then, is the first and the most important symptom, followed closely by nausea and vomiting; the increased pulse rate and rise in temperature are evident in exact proportion to the exact proportion to the pathological progress in the appendix or peritoneum; the rigidity of the abdominal wall follows this damage and is Nature's attempt to protect a peritoneum that either has already become inflamed or is rapidly becoming so.

The gravity of an acute inflammation of the appendix is due not so much to what damage may be done to this vestigial organ, as to the dangers of local, general, or diffuse peritonitis caused by the rupture into the free abdominal cavity of its highly infected contents.

Undisputed cases of catarrhal appendicitis have been reported by eminent medical men as cured and there is no doubt that little if any pathology would be in evidence if the appendix could be examined, but there is usually some damage done to the mucous lining of the organ that perverts the normal currents of the appendix; the lumen is either lessened or perhaps obliterated, or there is an extravasation of blood or serum into the canal which unites with the inorganic substances of secretion, and promotes cohesion which forms a nucleus for a concretion. The presence of this concretion, then, becomes a constant source of irritation and there at once arises the question of the equation of resistance and infective virulence. So long as the resistance is greater than the infective ability of the invading host, so long is the integrity of the part maintained; but with a lessened blood supply or lowered support from any cause the structure of the organ is compromised and degeneration or destruction takes place in exactly the proportion that the increased activity of the infective agent bears to the physical resistance of the part, be it much or little.

An appendix containing a concretion or having a stenosis of its lumen is, to a certain extent, in a constant state of hyperemia, and dissolution may take place in a very few hours. A local peritonitis producing the acute pain, the vomiting, increase in pulse rate, and rise in temperature with abdominal rigidity cannot be called early in the attack with anywhere near the security that one would like to feel in operating in these cases that have been under observation perhaps but a few hours, and which, according to the time standard, would be called early. When we find in any case adhesions, with local peritonitis of varying degree, an appendix containing pus, concretions, or even a stenosis of its lumen with a gangrenous mucosa, it surely is not early in the course of the disease, even if the presence of the latter has been known but a few hours, nor is such a case likely to give the promised prompt recovery we would like to expect.—*Medical Standard*, December, 1906.

